# Green Synthesis, Characterizations of Zinc Oxide Nanoparticles from Aqueous Leaf Extract of *Tridax procumbens* Linn. and Assessment of their Anti-Hyperglycemic Activity in Streptozoticin-Induced Diabetic Rats

**DOI:** 10.3390/ma15228202

**Published:** 2022-11-18

**Authors:** Syed S. Ahmed, Ali M. Alqahtani, Taha Alqahtani, Ali H. Alamri, Farid Menaa, Rupesh Kumar Mani, Bharathi D. R., Kunchu Kavitha

**Affiliations:** 1 Department of Pharmacology, Sri Adichunchanagiri College of Pharmacy, Adichunchanagiri University, BG Nagara, Mandya 571448, India; 2 Department of Pharmacology, College of Pharmacy, King Khalid University, Abha 62529, Saudi Arabia; 3 Department of Pharmaceutics, College of Pharmacy, King Khalid University, Abha 62529, Saudi Arabia; 4 Departments of Internal Medicine and Nanomedicine, Fluorotronics, Inc. & California Innovations Corporation, San Diego, CA 92037, USA; 5 Department of Pharmaceutics, NITTE College of Pharmaceutical Sciences, Bangalore 560064, India

**Keywords:** *Tridax procumbens*, streptozotocin, zinc oxide nanoparticles, antidiabetic, glycated hemoglobin, nanomedicine

## Abstract

Herein, zinc oxide nanoparticles (ZnO NPs) were greenly synthesized from *Tridax procumbens* aqueous leaf extract (TPE) and characterized physically (e.g., Fourier-transform infrared (FTIR) spectroscopy and scanning electron microscopy (SEM)) and biologically (test of their anti-diabetic activity). Anti-diabetic activities of TPE and TPE-derived ZnO NPs have been carried out in a streptozotocin (STZ)—induced diabetic rat model. Diabetes mellitus (DM) was induced with a single intraperitoneal dosage of the glucose analogue STZ (55 mg/Kg) known to be particularly toxic to pancreatic insulin-producing beta-cells. TPE and TPE-derived ZnO NPs were administered orally, once every day for 21 days in diabetic rats, at 100 and 200 mg/Kg, respectively. The standard antidiabetic medication, glibenclamide, was used as a control at a dose of 10 mg/Kg. Various parameters were investigated, including bodyweight (bw) variations, glycemia, lipidaemia, glycated hemoglobin (HbA1c), and histopathological alterations in the rat’s liver and pancreas. The TPE-mediated NPs were small, spherical, stable, and uniform. Compared to TPE and, to a lesser extent, glibenclamide, TPE-derived ZnO NPs lowered blood glucose levels considerably (*p* < 0.05) and in a dose-dependent manner while preventing body weight loss. Further, positive benefits for both the lipid profile and glycated hemoglobin were also noticed with TPE-derived ZnO NPs. The histopathological assessment revealed that synthesized TPE-derived ZnO NPs are safe, non-toxic, and biocompatible. At 200 mg/Kg/day, TPE-derived ZnO NPs had a more substantial hypoglycemic response than at 100 mg/Kg/day. Thus, in this first reported experimental setting, ZnO NPs biosynthesized from the leaf extract of *Tridax procumbens* exert more potent anti-diabetic activity than TPE and glibenclamide. We conclude that such a greenly prepared nanomaterial may be a promising alternative or complementary (adjuvant) therapy, at least to the current Indian’s traditional medicine system. Translational findings are prompted in human populations to determine the efficacy of these NPs.

## 1. Introduction

DM is a common metabolic condition marked by excessive blood glucose levels caused by insufficient insulin synthesis from the pancreatic β-cells or unresponsive body cells to insulin. Globally, many people have diabetes, and its number is increasing at an alarming rate. Diabetes kills 1.5 million people worldwide every year, and it is a prominent cause of death, according to the estimation of the World Health Organization (WHO) [1]. Its prevalence in 2010 was estimated at 285 million, accounting for 6.4% of the globe’s population. It is projected to rise by 438 million by 2030. Diabetes is on the track to become the world’s 7th largest cause of death, accounting for 3.3 percent of all deaths worldwide, according to the International Diabetes Federation [2].

To counteract the deleterious effects of this increasing threat to people and society, alternative and complementary therapies must be continuously developed. In economically poor countries and/or where traditional medicine is still practiced extensively, cost-effective, rationally designed solutions must be offered.

*Tridax procumbens* Linn. belongs to the family of Asteraceae (Common Name: coat buttons), a widespread flowering herb found throughout India [3]. The weed has been claimed to cure various ailments, including diarrhea, bronchial catarrh, hair loss, and dysentery. Indeed, this herb has potent properties, such as hypotensive, antimicrobial, anti-inflammatory, immunomodulatory, wound healing, and antiseptic [3]. Traditionally, this plant leaf powder has been used orally to treat diabetes. The medicinal properties of *T. procumbens* are explained by the presence of numerous phytocomponents, such as quercetin, sitosterol, β-sitosterol, luteolin, glycoside, dexamethasone, flavone, and glucotureolin [3].

Green or biogenic technologies are currently used to synthesize (via a bottom-up approach) metal NPs because they are eco-friendly, cost-effective, fast, simple, and safe. Indeed, such NP synthesis routes use negligeable chemicals, resulting in less pollution, lower costs, and energy requirements [4].

In general, NPs are suitable for a wide range of biological applications. Cells can easily take up NPs due to their ultra-small size with enhanced surface area, and this allows proper interaction with biomolecules to elicit specific biological responses. Zinc (Zn) is a trace metal that affects several enzymatic activities and cellular processes in the human body, including apoptosis, oxidative equilibrium, immunological function, metabolic regulation, and signal transduction. Zn supplementation has been found to positively impact glycemic control in diabetic patients. In addition, zinc also improves insulin signaling through several mechanisms, including increased insulin receptor phosphorylation, enhanced PI3K activity, and inhibition of glycogen synthase kinase-3 (GSK-3). Furthermore, this metal has been suggested to ameliorate diabetic complications, such as nephropathy and cardiomyopathy [5]. Various synthetic glucose-lowering medications are available nowadays. However, considering their adverse effects and expensive costs, medicinal plants have received a lot of interest in treating DM [6,7]. Interestingly, ZnO-NPs, a novel agent for delivering zinc, have great implications in many disease therapies, including DM [5].

Previous research on TPE showed a convincing hypoglycemic impact in alloxan-induced diabetic rats [3,5]; however, to the best of our knowledge, no studies have reported the antidiabetic effect of the green synthesis of TPE-mediated ZnO NPs. Thus, we decided to use STZ-induced diabetic rats to determine the potential antidiabetic activity of both TPE (traditionally used in India) and freshly biofabricated TPE-mediated ZnO NPs.

## 2. Materials and Methods

### 2.1. Plant Material

*Tridax procumbens* leaves were collected from different regions of B. G Nagara, Mandya district, Karnataka, India, during July–August 2021. The leaves were identified and authenticated by an experienced botanist at Maharani’s Science College for Women, Mysore, India. For future reference, the voucher/specimen number (#MGBH47) of the plant, deposited in the Herbarium, Botany Department of the University, has been provided to the Pharmacology department of Sri Adichunchanagiri College of Pharmacy, Mandya, India.

The collected healthy and fresh leaves were then thoroughly washed, shade dried, and coarse powdered using a mechanical grinder. The resulting powder was then utilized to prepare the plant extract.

#### 2.1.1. Preparation of Plant Extract

Aqueous leaf TPE was prepared in double distilled water (ddH_2_O) at a ratio of 1:10 (100 g TPE/1000 mL ddH_2_O) in a boiling flask kept for 15 min in a water bath at 60 °C. Then, the mixture was chilled and stirred for 20–30 min (using a magnetic stirrer) to enhance the extraction. The solution was filtered with the help of Whatman No.1 filter paper to remove the leaf debris. The clear extract filtrate was kept at 4 °C until use.

#### 2.1.2. Phytochemical Screening

The phytochemical screening and analysis were performed on the TPE to determine the presence of several phytoconstituents (e.g., alkaloids, polyphenols), according to a previously described procedure [8].

### 2.2. Preparation of ZnO NPs from TPE

For the photosynthesis of ZnO NPs, 100 mL of 1 mM zinc sulfate (ZnSO_4_) solution was first prepared. The TPE was then thoroughly mixed with a ZnSO4 solution (1:9). The mixture was incubated for four days at room temperature (RT), and the NP’s formation was visually monitored by UV-Vis spectroscopy (Shimadzu UV-1800, Carlsbad, CA, USA). Subsequently, the mixture was centrifuged at 5000 rpm for 15 min. The pellets were separated and resuspended in distilled water and centrifuged again under the same conditions. Eventually, the pellets were dried using a hot air oven until moisture was eliminated. The dried particles were collected and used in further experiments.

### 2.3. Physical Characterizations

#### 2.3.1. UV-Visible Spectroscopy

The generation of NPs by the bioreduction of metal ions from the salt solution/TPE mixture was monitored at 24-h intervals for four days. The produced ZnO NPs were subjected to UV-Vis spectroscopic examination (Shimadzu UV-1800, Carlsbad, CA, USA).

#### 2.3.2. Fourier Transform Infrared Spectroscopy (FTIR)

The functional groups found in the photosynthesized ZnO NPs. were determined using FTIR spectroscopy (Shimadzu 8400S, Tokyo, Japan). The sample was combined with KBr salt in the mid-IR range of frequency 400–4000 cm^−1^, and the measurements were made with a resolution of 2 cm^−1^ [4].

#### 2.3.3. Zeta Potential (ZP)

The ZP of the bioprepared ZnO NPs was detected by photon correlation spectroscopy (Zeta—Nano ZS 90, Malvern instruments Ltd., Malvern, UK) to detect the surface charge and stability.

#### 2.3.4. Scanning Electron Microscopy (SEM) Analysis

The surface morphology of the biogenic ZnO NPs was analyzed using SEM (ZEISS EVO 18 Research, Jena, Germany). The sample was simply dropped on top of the carbon-coated copper grid to form a thin layer. The excessive solution was eliminated by blotting paper and allowed to dry the film under a mercury lamp for five minutes.

#### 2.3.5. Particle Size Analysis

The average PS of the TPE-mediated ZnO NPs was analyzed using a particle size analyzer (PSA) (SALD-2300, Shimadzu, Columbia, MO, USA) [9].

### 2.4. Animals

Albino rats of 3 to 4 months old and either sex, weighing about 160–200 g, were procured from Vaarunya Biolabs Private Limited (CPCSEA Registration No. 2076/P O/RcBi Bt/S/19/C PCSEA), Bengaluru, Karnataka, India. Rats were kept in wide polyacrylic cages under standard husbandry settings (25 ± 2 °C temperature, 60–70% relative humidity, and a 12-h light/dark cycle). They were fed standard rat pellets with access to drinking water ad libitum. Experiments were conducted with the approval of both the Committee for the Purpose of Control and Supervision of Experiments on Animals (CPCSEA), India, and the Institutional Animal Ethical Committee (IAEC) of Sri Adichunchanagiri, College of Pharmacy, India, who authorized the experimental procedures (IAEC Approval No. SACCP-IAEC/2020-01/22).

### 2.5. In-Vivo Antidiabetic Activity of TPE and TPE-Mediated ZnO NPs

#### 2.5.1. Induction of Diabetes

A single intraperitoneal injection of freshly produced STZ (55 mg/Kg bw, Yarow Chem Products, Mumbai, Maharashtra, India) in 0.1 M citrate buffer (pH 4.5, Sisco Research Laboratories Pvt. Ltd. Taloja, Maharashtra, India) was used overnight in the starved rats to induce diabetes. To avoid STZ-induced hypoglycemic mortality, the rats were given a 20% glucose solution to consume for 24 h. One-week post-STZ injection, the development of diabetes was detected, and rats with fasting blood glucose levels of more than 200 mg/dL were selected for the study [10,11].

#### 2.5.2. Experimental Design

The rats (*n* = 42) were split into seven groups, each consisting of six individuals. All the test substances were suspended with distilled water (dH_2_O) in a vehicle containing 0.1 percent Carboxymethylcellulose (CMC, Sisco Research Laboratories Pvt. Ltd. Taloja, Maharashtra, India), and were given to all animals orally once a day for 21 days as follows:

Group I: Normal control (0.1% CMC suspension)

Group II: Diabetic control (received STZ 55 mg/Kg bw)

Group III: TPE (100 mg/Kg bw)

Group IV: TPE (200 mg/Kg bw) treatment

Group V: TPE-mediated ZnO NPs (100 mg/Kg bw)

Group VI: TPE-mediated ZnO NPs (200 mg/Kg bw)

Group VII: Glibenclamide (10 mg/Kg bw, Yarow Chem Products, Mumbai, Maharashtra, India)

#### 2.5.3. Body Weight (bw) Analysis

The bw of each rat was noted at an interval of 7 days from the beginning until the end of the experiment (i.e., 1st, 7th, 14th, and 21st day), using a balance (Docbel Industries, New Delhi, India).

#### 2.5.4. Blood Glucose Level Analysis

Diabetic rats were given glibenclamide, TPE, and TPE-mediated ZnO NPs for 21 days. To estimate the blood glucose level, the blood samples were obtained by rupturing the tail vein and determined on the 1st (0 h and 2nd h), 7th, 14th, and 21st days of therapy using an On Call Plus glucometer (ACON Biotech (Hangzhou) Co., Ltd., West Lake District, Hangzhou, China).

#### 2.5.5. Biochemical Analysis

The rats were slaughtered at the end of the experiment (on the 21st day) under anesthetic circumstances using ketamine hydrochloride 80 mg/Kg bw administered intraperitoneally. Blood samples were withdrawn through cardiac puncture and analyzed following previously described procedures [5,12,13]. Total cholesterol (TC), triglycerides (TG), high-density lipoprotein (HDL), low-density lipoprotein (LDL), and very low-density lipoprotein (VLDL) were the lipid profile parameters assessed. The glycated hemoglobin (Hb1Ac) was also estimated.

#### 2.5.6. Histopathological Analysis

The pancreas and liver were removed and washed continuously with phosphate-buffered saline (1× PBS, pH 7.4) and preserved by immersing in a 10% formalin solution and embedded in paraffin blocks. Five micrometer thick sections were prepared using a semi-automated rotator microtome and subjected to hematoxylin and eosin staining. Sections were imaged using an inverted biological microscope (FM-BM-B200, Fison Instruments Ltd., Glasgow G2 4JR, UK) [14].

#### 2.5.7. Statistical Analysis

Data were expressed as mean ± SEM, and differences between the groups were statistically determined by analysis of variance (ANOVA, GraphPad Prism version. 8.0.2, San Diego, CA, USA). *p*-values < 0.05 were considered statistically significant.

## 3. Results and Discussion

The phytochemical analysis of the aqueous leaf extract of *T. procumbens* Linn. revealed the presence of amino acids, phytosterols, carbohydrates, tannins, proteins, phenolic compounds, alkaloids, glycosides, flavonoids, and saponins in the leaf extract. However, triterpenoids were absent. The related data are summarized in Table 1.

### 3.1. Characterizations of TPE-Mediated ZnO NPs

#### 3.1.1. UV-Visible Spectroscopy

**Figure 1:**
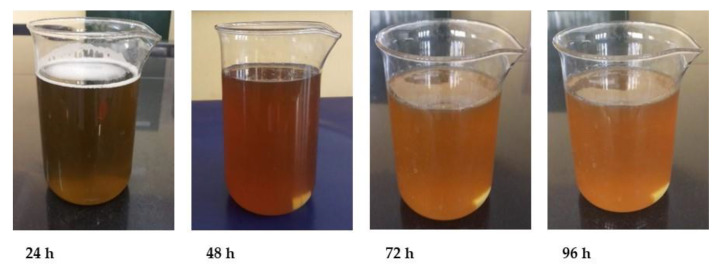
Gradual color change of the TPE-derived ZnO NP solution over time (for 4 days). A progressive shift in the color of the solution from green to yellow indicated the formation of ZnO NPs.

The biosynthesis of ZnO NPs was confirmed by a progressive shift in the color of the solution from green to yellow (Figure 1), which might be attributable to the surface plasmon resonance (SPR) of the produced NPs. In addition, TPE phytochemicals might play a key role in the bioreduction of Zn^2+^ ions to Zn0 ions and so in the synthesis of ZnO NPs [15]. Phytogenic synthesis of metal NPs represent advantages (e.g., cost-effectiveness, as an eco-friendly method) over physical and chemical methods [16,17].

**Figure 2:**
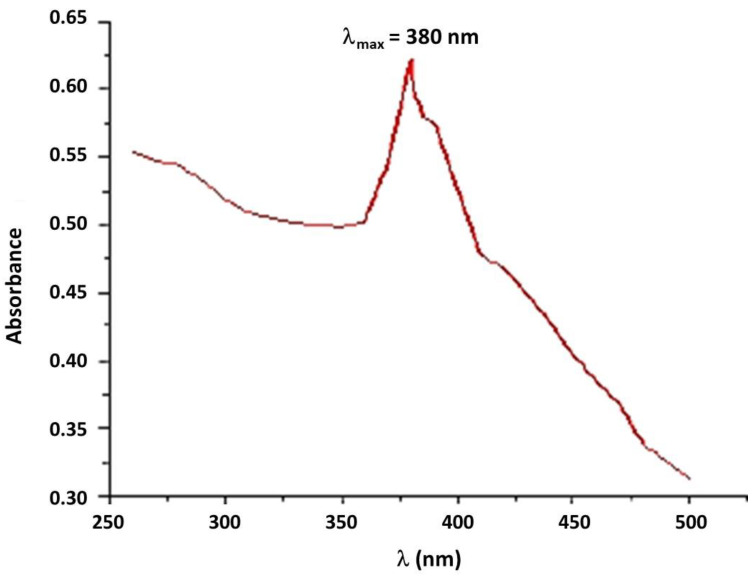
UV-visible spectrum of TPE-mediated ZnO NPs.

The ZnO NPs were analyzed using a UV-Vis Spectrophotometer at a wavelength range of 250 to 500 nm. The highest peak of absorbance observed at 380 nm (Figure 2) may indicate the probable formation of ZnO NPs [18].

#### 3.1.2. FTIR

**Figure 3:**
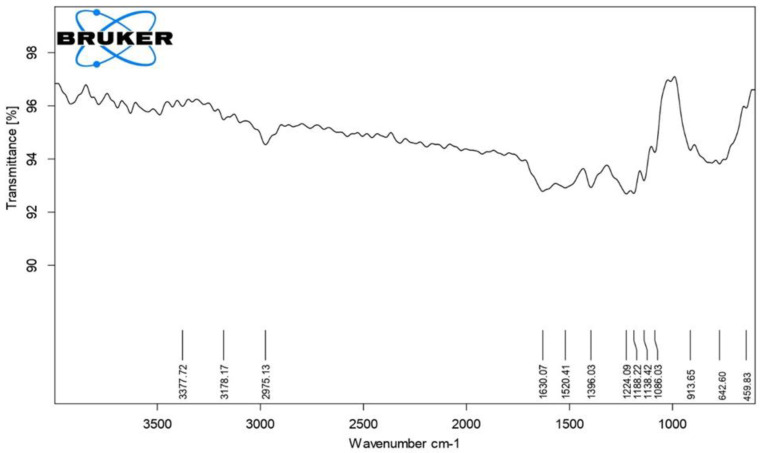
FTIR spectrum of TP-mediated ZnO NPs.

The FTIR spectrum of TPE-mediated ZnO NPs shows the presence of functional groups in the scan region of 4000–400 cm^−1^ (Table 2 and Figure 3). The O-H group is represented by the distinctive peak observed at 3377.72 cm^−1^. Alkanes C-H stretching vibration mode is shown by the peaks at 2975 cm^−1^. Among the other peaks, the one at 1630.07 cm^−1^ represents primary amines, and the one at 1520 cm^−1^ depicts nitro compounds. The peaks at 1224 cm^−1^ and 1138 cm^−1^, represents the presence of ester, carboxylic acid, ether, and alcohols. The peaks at 400–600 cm^−1^ are attributed to the metal-oxygen groups. At bending vibration, ZnO binding is shown by a peak at 459 cm^−1^. The FTIR spectra revealed that phytochemicals, such as flavonoids, proteins, phenolics, and alkaloids, might contribute to the production and stability of biogenically reduced ZnO NPs [19].

#### 3.1.3. ZP

**Figure 4:**
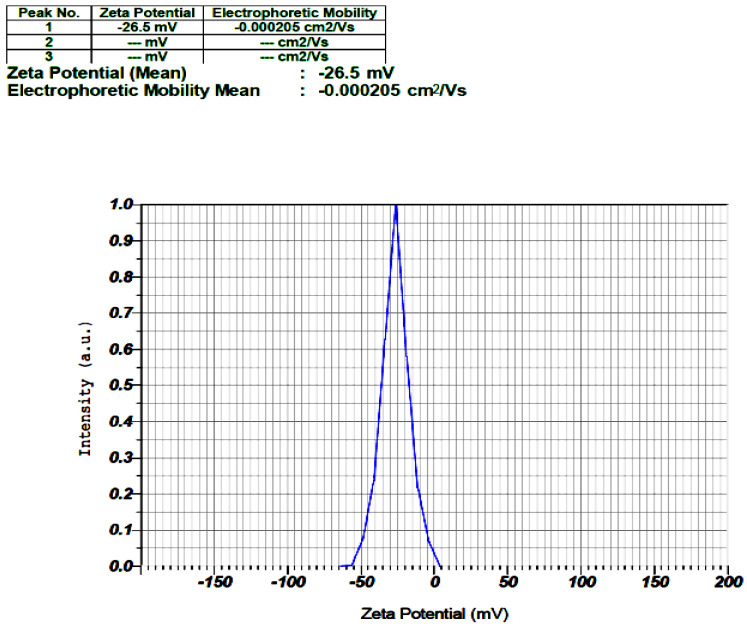
Zeta potential (mean) of TPE-mediated ZnO NPs. The negativity and distribution of ZP indicate relatively good stability, unaggregated NPs, and a suitable uniform distribution.

ZP is an essential metric for understanding and forecasting the electrostatic potential at the surface of NPs. The level of zeta potential indicates the colloidal stability of nanoparticles. Particles with large positive and negative zeta potential values repel each other, and no nanoparticle aggregation occurs. NPs with zeta potential values greater than +25 mV or less than −25 mV often have a high degree of stability. Lower ZP values cause aggregation owing to van der Waals interparticle attraction [20]. The ZP of ZnO NPs was found to be −26.5 mV, indicating a homogeneous distribution with possible long-term stability (Figure 4). Indeed, these findings show that NPs are relatively stable and may be prevented from self-aggregation.

#### 3.1.4. SEM and PS Analyses

**Figure 5:**
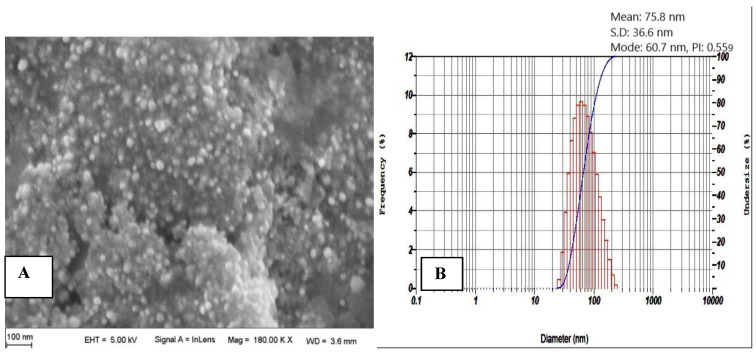
(**A**). SEM micrographs of TPE-mediated ZnO NPs. viewed at 180 K × magnifications with a 100 nm scale, (**B**). PSD of TPE-mediated ZnO NPs. The data indicate NPs < 100 nm and a PI < 0.7 demonstrating relatively small NPs and uniform PSD.

As a step forward, SEM was used to examine the surface morphology of the optimized TPE-mediated ZnO NPs. The data revealed NPs with a distinct spherical form and a smooth surface (Figure 5A). PSA shows an acceptable uniformity in particle size distribution (PSD) when dynamic light scattering (DLS) is used and based on the relatively low polydispersity (PDI or PI) of 0.56 (fits the range of 0.3–0.7). The histograms of the PSA (Figure 5B) indicate that the average size of the nanoparticles obtained is 75.8 ± 36.6 nm.

### 3.2. Evaluation of the Anti-Diabetic Activity of TPE versus TPE-Mediated ZnO NPs

#### 3.2.1. Blood Glucose Level

**Figure 6:**
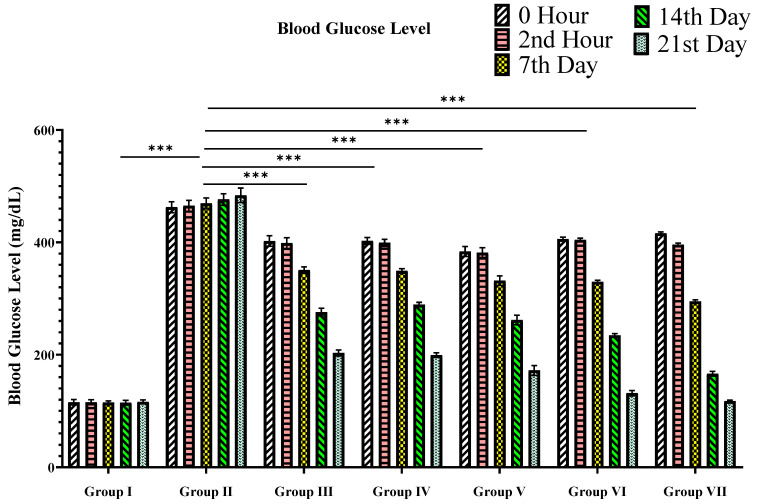
Graphical representation of the effects of TPE and TPE-ZnO NPs on glycemia (mg/dL) in STZ-induced diabetic rats. Group I: Normal control (0.1% CMC suspension); Group II: Diabetic control (received STZ 55 mg/Kg bw); Group III: TPE (100 mg/Kg bw); Group IV: TPE (200 mg/Kg bw) treatment; Group V: TPE-mediated ZnO NPs (100 mg/Kg bw); Group VI: TPE-mediated ZnO NPs (200 mg/Kg bw); Group VII: Glibenclamide (10 mg/Kg bw). Values are expressed as mean ± SEM, *n* (no. of animals in each group) = 6.; *p* < 0.001 (***) compared to diabetic animals = Group II (two-way ANOVA followed by a Dunnett’s *t*-test). *p*-values < 0.05 were considered statistically significant.

As represented in Table 3 and Figure 6, when TPE and TPE-derived ZnO NPs at doses of 100 and 200 mg/Kg bw were administered for 21 days in STZ-induced diabetic albino rats, a significant dose-dependent drop was confirmed in blood glucose levels compared to the reference drug glibenclamide (10 mg/Kg). When compared to TPE alone, TPE-derived ZnO NPs substantially lowered blood glucose levels (*p*-values < 0.05).

#### 3.2.2. Body Weight

**Figure 7:**
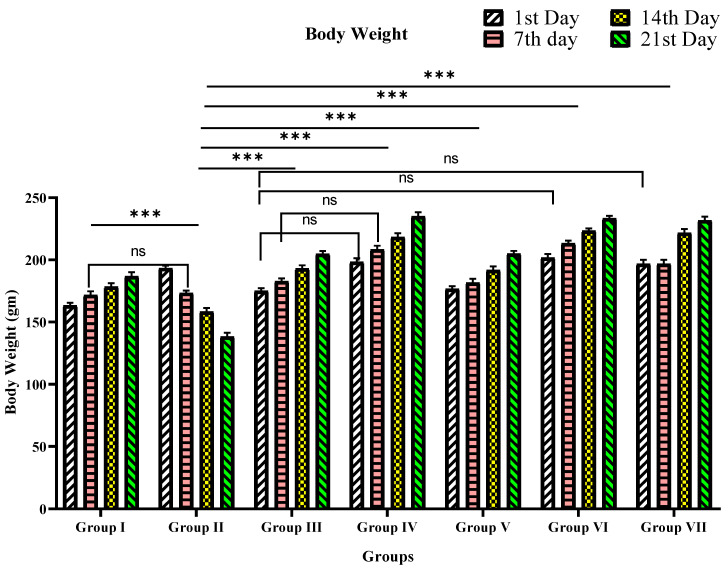
Graphical representation of TPE and TPE-ZnO NPs’ effects on body weight (g) in STZ-induced diabetic rats. Group I: Normal control (0.1% CMC suspension); Group II: Diabetic control (received STZ 55 mg/Kg bw); Group III: TPE (100 mg/Kg bw); Group IV: TPE (200 mg/Kg bw) treatment; Group V: TPE-mediated ZnO NPs (100 mg/Kg bw); Group VI: TPE-mediated ZnO NPs (200 mg/Kg bw); Group VII: Glibenclamide (10 mg/Kg bw). Values are expressed as mean ± SEM, (*n* = 6); *p* < 0.12 (ns), *p* < 0.001 (***) compared to diabetic animals/Group II (two-way ANOVA followed by a Dunnett’s *t*-test). *p*-values < 0.05 were considered statistically significant; ns stands for non-significant.

As shown in Table 4 and Figure 7, the bw has been reduced progressively from the 1st to 21st day in diabetic rats. However, bw loss was prevented after treating diabetic rats with glibenclamide, TPE, and TPE-ZnO NPs. The bw was prominently regained in the TPE-ZnO NPs compared to TPE alone.

### 3.3. Blood Lipid Level

**Figure 8:**
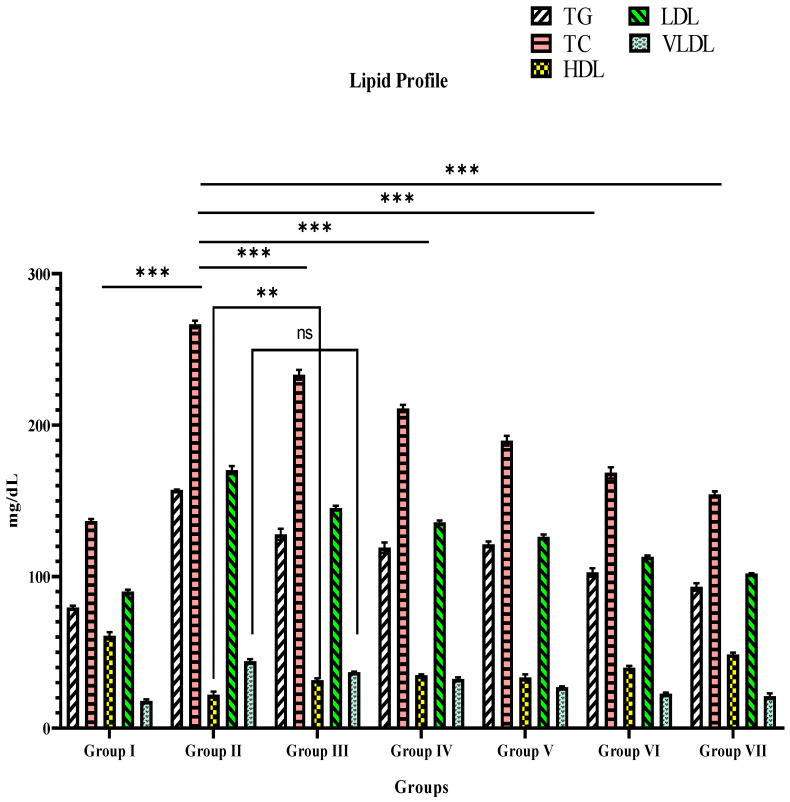
Graphical representation of TPE and TPE-ZnO NPs’ effects on lipidemia (mg/dL) in STZ-induced diabetic rats. Group I: Normal control (0.1% CMC suspension); Group II: Diabetic control (received STZ 55 mg/Kg bw); Group III: TPE (100 mg/Kg bw); Group IV: TPE (200 mg/Kg bw) treatment; Group V: TPE-mediated ZnO NPs (100 mg/Kg bw); Group VI: TPE-mediated ZnO NPs (200 mg/Kg bw); Group VII: Glibenclamide (10 mg/Kg bw). Values are expressed as mean ± SEM, (*n* = 6); *p* < 0.12 (ns), *p* < 0.002 (**), *p* < 0.001 (***) compared to diabetic animals/Group II (two-way ANOVA followed by a Dunnett’s *t*-test). *p*-values < 0.05 were considered statistically significant.

**Figure 9:**
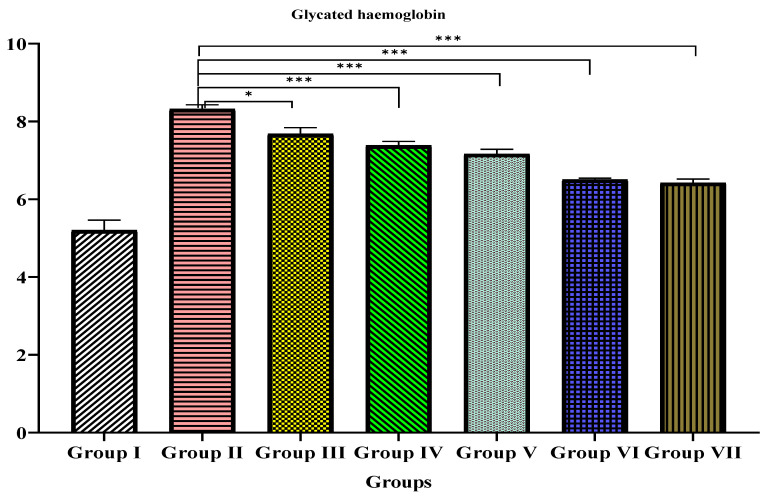
In STZ-induced diabetic rats, the effects of TPE, TPZnO NPs, and glibenclamide on glycated hemoglobin. Group I: Normal control (0.1% CMC suspension); Group II: Diabetic control (received STZ 55 mg/Kg bw); Group III: TPE (100 mg/Kg bw); Group IV: TPE (200 mg/Kg bw) treatment; Group V: TPE-mediated ZnO NPs (100 mg/Kg bw); Group VI: TPE-mediated ZnO NPs (200 mg/Kg bw); Group VII: Glibenclamide (10 mg/Kg bw). Values are expressed as mean ± SEM, (*n* = 6); *p* < 0.033 (*), *p* < 0.001 (***) compared to diabetic animals/Group II (two-way ANOVA followed by a Dunnett’s *t*-test).

For 21 days of therapy, diabetic control rats had higher levels of TC, TG, LDL, and VLDL and lower HDL levels than normal control rats (Table 5 and Figure 8). These parameters were reversed in glibenclamide (10 mg/Kg bw), TPE, and TPE-mediated ZnO NP-treated groups, and the effect was higher (*p* < 0.001 (***)) in TPE-derived ZnO NPs compared with TPE (*p* < 0.001 (***)) and glibenclamide (*p* < 0.001 (***)) (Table 5 and Figure 9). This demonstrates a considerable dose-dependent improvement in the lipid profile of TPE-mediated ZnO NPs.

### 3.4. Glycated Hemoglobin

**Figure 10:**
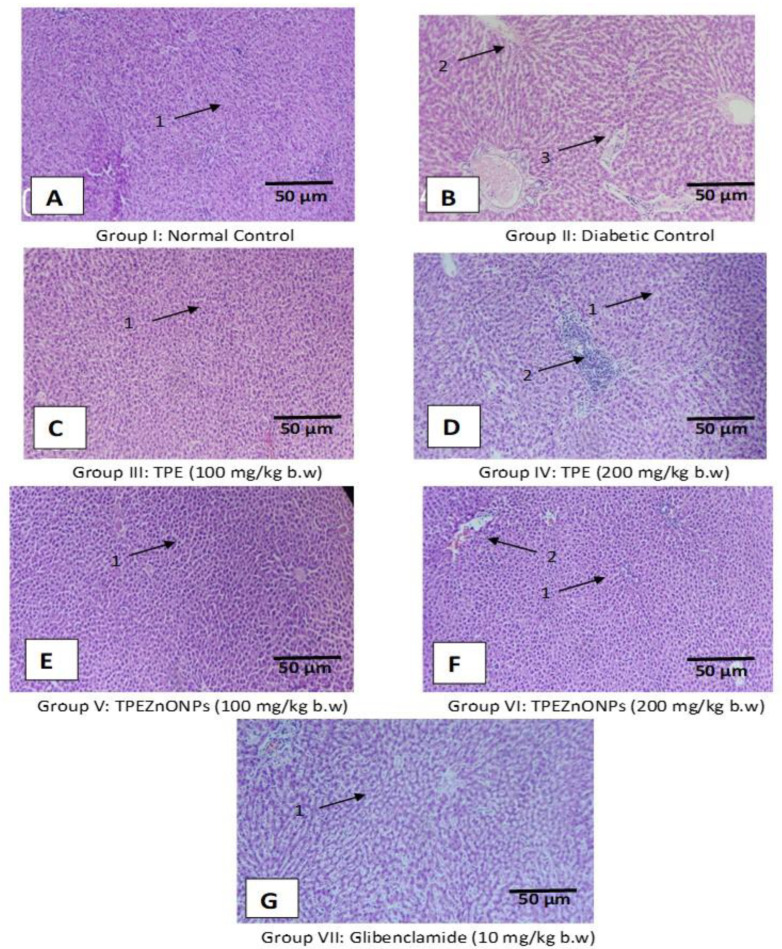
The liver section of histology stained with hematoxylin and eosin (Scale bar 50 μm, 100× magnification). (**A**) Group I: Normal control, (**B**) Group II: Diabetic control (received STZ), (**C**) Group III: TPE (100 mg/Kg bw), (**D**) Group IV: TPE (200 mg/Kg bw), (**E**) Group V: TPE-derived ZnO NPs (100 mg/Kg bw), (**F**) Group VI: TPE-derived ZnO NPs (200 mg/Kg bw), (**G**) Group VII: Glibenclamide (10 mg/Kg bw). Where 1: Normal hepatocyte, 2: mild periportal inflammation, 3: periportal fatty infiltration.

Compared to the normal control (Group I), the diabetic control rats (Group II) showed significantly elevated (*p* < 0.001) concentration levels of HbA1c on the 21st day of treatment (Table 6 and Figure 9). Compared to Group 2, the glycated hemoglobin level was decreased in the TPE (Groups III and IV), TPE-derived ZnO NPs (Groups V and VI), and glibenclamide (Group VII)-treated groups. (Table 6 and Figure 10) This shows a significant dose-dependent effect on HbA1C%.

### 3.5. Histopathological Examination

#### 3.5.1. Liver Cells

In the normal control rats, the histoarchitecture of the liver was normal (Figure 10A). The sinusoids and hepatic parenchyma were expected, with normal Kupffer cells (KC) distribution in contrast to the diabetic control rat’s liver, which showed necrotic alterations, dilatation of liver sinusoids (LS), activation of KC, cytoplasmic vacuolization (CV) of hepatocytes, mild periportal inflammation, and periportal fatty infiltration (Figure 10B). Post-treatment of diabetic rats with standard and test drugs showed varying degrees of improvement (Figure 10C–G). The TPE-treated groups’ liver histoarchitecture was regular, with only minimal necrobiotic alterations (NT) and degeneration (FCH) (Figure 10C,D). There was also a slight increase in the quantity and dispersion of KC and a minor dilatation of the blood sinusoids (BS). The TPE-derived ZnO NP group’s liver revealed marked sinusoidal dilatation, minor necrotic alterations, and a congested CV in the presence of KC (Figure 10E,F). The glibenclamide-treated group’s liver revealed modest degenerative alterations and a moderate dilatation of the hepatic sinusoid dilatation with a relatively larger number of KC (Figure 10G).

#### 3.5.2. Pancreatic Cells

**Figure 11:**
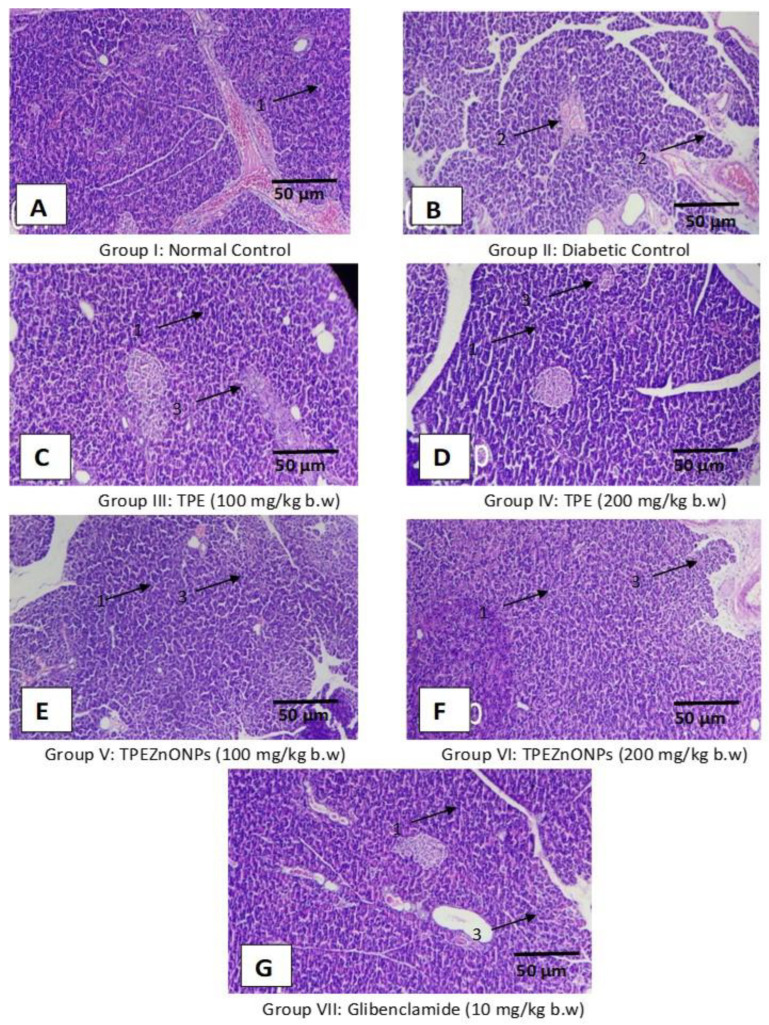
The pancreatic section of histology stained with hematoxylin and eosin (Scale bar 50 μm, 100× magnification).(**A**) Group I: Normal control, (**B**) Group II: Diabetic control (received STZ), (**C**) Group III: TPE (100 mg/Kg bw), (**D**) Group IV: TPE (200 mg/Kg bw), (**E**) Group V: TPE-derived ZnO NPs (100 mg/Kg bw), (**F**) Group VI: sTPE-derived ZnO NPs (200 mg/Kg bw), (**G**) Group VII: Glibenclamide (10 mg/Kg bw). Where 1: Beta cells Islets, 2: Depletion of beta cells, 3: Formation of Islets.

In the normal control rats, histological architecture of the pancreas was expected (Figure 11A). It was in the form of an acinar structure with normal islets of Langerhans. The diabetic control group’s pancreas revealed a significant reduction in islets of Langerhans (IL) and an acini atrophy, vacuolar degeneration, and necroptosis (Figure 11B). In the Glibenclamide-treated group, IL returned to a standard size, with normal acinar cells and mild necrotic changes (Figure 11G). The pancreas of the TPE-treated groups revealed a modest reconstitution of IL cells with minimal necrotic alterations (Figure 11C,D). With the restoration of IL and minimal necrotic alterations, the pancreas of the TPZnO NP-treated groups seemed normal (Figure 11E,F). In both the standard and test drug-treated groups, improvements in pancreatic cells were observed (Figure 11C–G).

## 4. Conclusions

We showed that the phytoconstituents present in *Tridax procumbens* leaf extract may play a decisive role in the biogenic production of ZnO NPs. UV-Visible Spectroscopy, FTIR, SEM, Zeta Potential, and PSA confirmed the lucrative production of zinc-loaded nanoparticles. Both extracts, as well as nanoparticles, showed a significant hypoglycemic response in a dose-dependent manner. However, the nanoparticles proved higher anti-diabetic activity as compared to the extract. This might be due to the unique property of a larger surface area-to-volume ratio, which helps in better drug delivery than bulk drugs. Glycemic control in diabetic individuals has been demonstrated to improve with zinc supplementation. The green synthesized ZnO NPs are chemically stable and cost effective. Cells can easily take up it due to its ultra-small size and enhanced surface area. This allows proper interaction with biomolecules to elicit specific biological responses. Hence, *Tridax procumbens*-zinc oxide nanoparticles can be a better remedy for treating DM after transforming it into a suitable dosage form.

## Figures and Tables

**Table 1:** Phytochemical analysis of aqueous leaf TPE.

Serial #	Phytochemical Compound Test		Result
1.	Carbohydrates	Molisch’s test	+
		Fehling’s test	+
		Barfoed’s test	+
2.	Test of starch		+
3.	Proteins and amino acids	Million’s test	+
		Biuret test	+
		Ninhydrin test	+
4.	Phenolic compounds and tannins	Ferric chloride test	+
		Test with Lead acetate Solution	+
		Gelatin test	+
5.	Phytosterols	Salkowski test	+
		Libermann–Burchards test	+
6.	Test for fixed oils and fats	Spot test	+
		Saponification	+
7.	Test for alkaloids	Mayer’s test	+
		Dragendroff’s test	+
		Wagner’s test	+
		Hager’s test	+
8.	Test for glycosides	Legal’s test	+
		Balget’s test	+
		Borntrager’s test	+
		Modified Borntrager’s test	+
9.	Test for flavonoids	Ferric chloride test	+
		Shinoda’s test	+
		Fluorescence test	+
		Reaction with alkali and acid	+
		Zinc, HCl reduction test	+
		Test for Flavonoids	+
10.	Test for saponins	+	
11.	Test for triterpenoids	-	

+ = Present, - = Absent.

**Table 2:** FTIR assignments of functional groups observed in TPE-mediated ZnO NPs.

FTIR Assignment Number	Frequency (cm^−1^)	Standard Frequency (cm^−1^) Range	Bond	Functional Groups
1	3377.72	3500–3200	O-H stretch H-bonded	Alcohols, phenols
		3400–3250	N-H stretch	Primary and secondary amines, amides
2	2975.13	3000–2850	C-H stretch	Alkanes
3	1630.07	1650–1580	N-H bend	Primary amines
4	1520.41	1550–1475	N-O asymmetric stretch	Nitro compounds
5	1224.09	1320–1000	C-O stretch	Alcohols, carboxylic acid, ester, ethers
6	1138.42	1320–1000	C-O stretch	Alcohols, carboxylic acid, ester, ethers
7	1086.03	1250–1020	C-N stretch	Aliphatic amines
8	913.65	950–910	O-H bend	Carboxylic acids
9	642.60	690–515	C-Br stretch	Alkyl halides

**Table 3:** Effects of TPE and TPE-ZnO NPs on blood glucose levels (mg/dL) in STZ-induced diabetic rats.

Groups	Blood Glucose Levels (mg/dL). Mean ± SEM Is Specified				
	1st Day		7th Day	14th Day	21st Day
	0 h	2nd h			
I	115.5 ± 4.8 ***	115.5 ± 4.5 ***	114.8 ± 3.2 ***	114.7 ± 4.2 ***	116 ± 3.5 ***
II	462.5 ± 9.7	465 ± 9.8	469.3 ± 9.8	476.3 ± 10.2	483.8 ± 12.8
III	402.5 ± 9.4 ***	398.7 ± 9.7 ***	350.5 ± 5.8 ***	275.7 ± 6.9 ***	203.3 ± 5.3 ***
IV	402.7 ± 5.9 ***	399.2 ± 6.2 ***	349.3 ± 4.0 ***	289.3 ± 4.0 ***	199.3 ± 4.0 ***
V	383.7 ± 8.9 ***	381.8 ± 8.6 ***	331.8 ± 8.6 ***	261.8 ± 8.6 ***	172 ± 8.7 ***
VI	406 ± 3.4 ***	404.5 ± 2.8 ***	329.8 ± 2.7 ***	234.5 ± 3.2 ***	131.5 ± 4.8 ***
VII	416.0 ± 2.7 ***	396.0 ± 2.7 ***	294.7 ± 3.2 ***	166.3 ± 4.2 ***	117.5 ± 1.9 ***

Group I: Normal control (0.1% CMC suspension); Group II: Diabetic control (received STZ 55 mg/Kg bw); Group III: TPE (100 mg/Kg bw); Group IV: TPE (200 mg/Kg bw) treatment; Group V: TPE-mediated ZnO NPs (100 mg/Kg bw); Group VI: TPE-mediated ZnO NPs (200 mg/Kg bw); Group VII: Glibenclamide (10 mg/Kg bw). Values are expressed as mean ± SEM, *n* (no. of animals in each group) = 6; *p* < 0.12 (ns), *p* < 0.001 (***) compared to diabetic animals = Group II (two-way ANOVA followed by a Dunnett’s *t*-test). *p*-values < 0.05 were considered statistically significant.

**Table 4:** Graphical representation of TPE and TPE-ZnO NPs’ effects on body weight (g) in STZ-induced diabetic rats.

Group	Mean Body Weight (g) (Mean ± SEM)			
	1st Day	7th Day	14th Day	21st Day
I	163.3 ± 2.1 ***	171.7 ± 3.0 ns	178.3 ± 3.0 ***	186.7 ± 3.3 ***
II	193.3 ± 2.1	173.3 ± 2.1	158.3 ± 3.0	138.3 ± 3.0
III	175.0 ± 2.2 ***	182.8 ± 2.3 ns	193 ± 2.6 ***	204.7 ± 2.4 ***
IV	198.3 ± 3.0 ns	208.3 ± 3.0 ***	218.3 ± 0.0 ***	235.0 ± 3.4 ***
V	176.7 ± 2.1 ***	181.7 ± 3.0 ns	191.7 ± 3.0 ***	205.0 ± 2.2 ***
VI	201.7 ± 3.0 ns	213.3 ± 2.8 ***	223.3 ± 2.1 ***	233.3 ± 2.1 ***
VII	196.7 ± 3.3 ns	196.7 ± 3.3 ***	221.7 ± 3.0 ***	231.7 ± 3.0 ***

Group I: Normal control (0.1% CMC suspension); Group II: Diabetic control (received STZ 55 mg/Kg bw); Group III: TPE (100 mg/Kg bw); Group IV: TPE (200 mg/Kg bw) treatment; Group V: TPE-mediated ZnO NPs (100 mg/Kg bw); Group VI: TPE-mediated ZnO NPs (200 mg/Kg bw); Group VII: Glibenclamide (10 mg/Kg bw). Values are expressed as mean ± SEM, (*n* = 6); *p* < 0.12 (ns), *p* < 0.001 (***) compared to diabetic animals/Group II (two-way ANOVA followed by a Dunnett’s *t*-test). *p*-values < 0.05 were considered statistically significant; ns stands for non-significant.

**Table 5:** Effects of TPE and TPE-ZnO NPs on blood lipid levels (md/dL) in STZ-induced diabetic rats.

Groups	Biochemical Parameters (mg/dL)				
	TG	TC	HDL	LDL	VLDL
I	79.50 ± 1.3 ***	136.50 ± 1.5 ***	60.83 ± 2.4 ***	90.0 ± 1.3 ***	17.83 ± 1.1 ***
II	157.0 ± 0.3	266.5 ± 2.4	22.1 ± 2.0	170.3 ± 2.6	44.00 ± 1.3
III	127.8 ± 3.6 ***	233.3 ± 3.3 ***	31.6 ± 1.1 **	145.2 ± 1.5 ***	37.00 ± 0.3 ns
IV	119 ± 3.0 ***	211.0 ± 2.4 ***	35.0 ± 0.6 ***	135.8 ± 1.2 ***	32.50 ± 1.1 ***
V	121.2 ± 1.8 ***	189.7 ± 3.1 ***	33.5 ± 1.9 ***	126.2 ± 1.5 ***	27.00 ± 0.5 ***
VI	102.7 ± 2.8 ***	168.5 ± 3.7 ***	39.8 ± 1.2 ***	113.0 ± 0.8 ***	22.67 ± 0.8 ***
VII	93.17 ± 2.4 ***	154.2 ± 1.9 ***	48.5 ± 1.3 ***	101.8 ± 0.30 ***	21.17 ± 1.9 ***

Group I: Normal control (0.1% CMC suspension); Group II: Diabetic control (received STZ 55 mg/Kg bw); Group III: TPE (100 mg/Kg bw); Group IV: TPE (200 mg/Kg bw) treatment; Group V: TPE-mediated ZnO NPs (100 mg/Kg bw); Group VI: TPE-mediated ZnO NPs (200 mg/Kg bw); Group VII: Glibenclamide (10 mg/Kg bw). Values are expressed as mean ± SEM, (*n* = 6); *p* < 0.12 (ns), *p* < 0.002 (**), *p* < 0.001 (***) compared to diabetic animals/Group II (two-way ANOVA followed by a Dunnett’s *t*-test). *p*-values < 0.05 were considered statistically significant; ns stands for non-significant.

**Table 6:** In STZ-induced diabetic rats, the effects of TPE, TPZnO NPs, and glibenclamide on glycated hemoglobin.

Group	Glycated Haemoglobin (HbA1c)%
I	5.2 ± 0.2
II	8.317 ± 0.1 ***
III	7.68 ± 0.1 *
IV	7.383 ± 0.1 ***
V	7.162 ± 0.1 ***
VI	6.505 ± 0.03 ***
VII	6.421 ± 0.09 ***

Group I: Normal control (0.1% CMC suspension); Group II: Diabetic control (received STZ 55 mg/Kg bw); Group III: TPE (100 mg/Kg bw); Group IV: TPE (200 mg/Kg bw) treatment; Group V: TPE-mediated ZnO NPs (100 mg/Kg bw); Group VI: TPE-mediated ZnO NPs (200 mg/Kg bw); Group VII: Glibenclamide (10 mg/Kg bw). Values are expressed as mean ± SEM, (*n* = 6); *p* < 0.033 (*), *p* < 0.001 (***) compared to diabetic animals/Group II (One-way ANOVA followed by a Dunnett’s *t*-test). *p*-values < 0.05 were considered statistically significant. Group II was also compared to Group I.

## Data Availability

Data that support the findings of this study are available from the corresponding author upon reasonable request.
